# Association between the National Cancer Screening Programme (NSCP) for gastric cancer and oesophageal cancer mortality

**DOI:** 10.1038/s41416-020-0883-x

**Published:** 2020-05-13

**Authors:** Jie-Hyun Kim, Kyung-Do Han, Jung Kuk Lee, Hyun-Soo Kim, Jae Myung Cha, Sohee Park, Joo Sung Kim, Won Ho Kim, for the Big Data Research Group (BDRG) of the Korean Society of Gastroenterology (KSG)

**Affiliations:** 1 Department of Internal Medicine, Yonsei University College of Medicine, Seoul, Korea; 2 Department of Biostatistics, The Catholic University, Seoul, Korea; 3 Department of Biostatistics, Yonsei University Wonju College of Medicine, Wonju, Korea; 4 Department of Internal Medicine, Yonsei University Wonju College of Medicine, Wonju, Korea; 5 Department of Internal Medicine, Kyung Hee University School of Medicine, Seoul, Korea; 6 The Department of Biostatistics, Graduate School of Public Health, Yonsei University, Seoul, Korea; 7 Department of Internal Medicine, Seoul National University School of Medicine, Seoul, Korea

**Keywords:** Population screening, Cancer screening

## Abstract

**Background:**

The aim was to evaluate whether this gastric cancer-screening programme was effective in reducing oesophageal cancer mortality.

**Methods:**

A population-based retrospective cohort study was conducted using the Korean National Cancer Screening Programme (NCSP) database. The study cohort comprised 16,969 oesophageal cancer patients who had been diagnosed in 2007–2014. We analysed the association between the history of NSCP for gastric cancer and oesophageal cancer mortality.

**Results:**

Compared with never-screened subjects, ever-screened subjects had an overall HR for oesophageal cancer mortality of 0.647 (95% CI, 0.617–0.679). According to the time interval since screening, the HRs of death were 0.731 (95% CI, 0.667–0.801) for 6–11 months, 0.635 (95% CI, 0.594–0.679) for 12–23 months, 0.564 (95% CI, 0.522–0.610) for 24–35 months and 0.742 (95% CI, 0.679–0.810) for ≥36 months. According to the last screening modality, the HRs of death were 0.497 (95% CI, 0.464–0.531) for upper endoscopy, and 0.792 (95% CI, 0.749–0.838) for UGIS. Upper endoscopy reduced the mortality consistently in all age groups over 50 years, whereas UGIS could not.

**Conclusion:**

The NCSP for gastric cancer was effective in reducing the mortality of oesophageal cancer, and upper endoscopy was superior to UGIS.

## Background

Oesophageal cancer (EC) is the eighth most common cancer and the sixth leading cause of cancer-related deaths worldwide.^1^ In Korea, EC is the tenth most common cause of cancer-related death.^2^ Although the prognosis of EC is considered quite poor because of rapid growth and early metastasis, the survival rate is dependent on the stage: the 5-year survival rate of early-stage EC has been reported to be ~60–80%, compared with a 1-year survival rate of <20% in unresectable or metastatic EC at time of diagnosis.^3,4^ Thus, early detection of EC is very important to improve prognosis. However, there are no global screening recommendations for EC. In particular, nationwide screening recommendations are difficult in nations with a low incidence of EC, like Korea.

In Korea, the National Cancer Screening Programme (NCSP) for gastric cancer was launched in 1999 to provide gastric cancer screening for individuals aged ≥40 years.^5,6^ The NCSP recommends biennial gastric cancer screening for men and women aged 40 years or older, by either upper gastrointestinal series (UGIS) or upper endoscopy.^7^ Despite having several limitations,^8^ the NCSP was recently reported to be effective in reducing gastric cancer mortality.^6^ According to that study, however, the effect of NCSP on reduction of gastric cancer mortality was observed only in those 40–74 years, not ≥75 years.

Examinations for gastric cancer can also detect EC, so screening programmes for gastric cancer can affect the prognosis of EC. However, no study has evaluated whether screening programmes for gastric cancer help to improve the prognosis of EC. Therefore, we evaluated whether the NCSP for gastric cancer can be effective to reduce EC mortality. We hypothesise that the current gastric cancer-screening programme is valid for improving the prognosis of EC as well as gastric cancer.

## Methods

### Study population

This population-based retrospective cohort study was conducted using data maintained by the NCSP since 2002 in Korea. Participants in the NCSP can choose to undergo either upper endoscopy or UGIS, depending on their personal preference. In 2002, National Health Insurance (NHI) beneficiaries in the lower 20% income bracket were eligible for the programme. In 2003 and 2004, the NCSP expanded its target population to the lower 30% income bracket, and in 2005, NHI beneficiaries in the lower 50% bracket were deemed eligible.

The study cohort consisted of 16,969 EC patients who had been diagnosed in 2007–2014. EC was defined using the International Classification of Diseases, 10th revision (ICD-10) codes (C15.0–15.5, C15.8 and C15.9). To assess the validity of defining EC using ICD-10, the data with codes (C15.0–15.5, C15.8 and C15.9) were extracted and analysed at Gangnam Severance Hospital between 2007 and 2014. All subjects with those codes (C15.0–15.5, C15.8 and C15.9) were considered to have EC. Furthermore, the number of subjects with EC defined using ICD-10 in our study was similar to that of the Korea Central Cancer Registry.^9^ According to that report,^9^ there were 2382 new cases of EC in 2013, which is similar to our data (2281 in 2013, Table 1).

**Table 1:** Baseline characteristics of study cohort.

Variables	*N* (%)
Year of diagnosis for oesophageal cancer	
2007	1845 (10.87)
2008	2043 (12.04)
2009	2022 (11.92)
2010	2124 (12.52)
2011	2098 (12.36)
2012	2250 (13.26)
2013	2281 (13.44)
2014	2306 (13.59)
Age (years, mean ± SD)	65.12 ± 9.92
Age group (years)	
<65	7610 (44.85)
65–74	6399 (37.71)
≥75	2960 (17.44)
Male	15136 (89.20)
Body mass index (BMI, mean ± SD)	22.85 ± 3.09
Waist circumference (mean ± SD)	82.71 ± 8.41
Comorbidity	
Diabetes mellitus	6091 (35.89)
Heart disease	9467 (55.79)
Cerebrovascular disease	4477 (26.38)
Liver disease	5455 (32.15)
Pulmonary disease	8891 (52.40)
Chronic kidney disease	300 (1.77)
Income level	
High	4203 (24.77)
Middle	7526 (44.35)
Low	5240 (30.88)
Proton pump inhibitor use	7043 (86.54)
Ever-screened	8666 (51.07)
Number of screening during 5 years before diagnosis	
None	8303 (48.93)
1	4542 (26.77)
≥2 or over	4124 (24.30)
Time interval since the last screening (month)	
None	8303 (48.93)
6–11	1376 (8.11)
12–23	3273 (19.29)
24–35	2460 (14.50)
≥36 or over	1557 (9.18)
The last screening method	
None	8303 (48.93)
Upper endoscopy	4190 (24.69)
Upper gastrointestinal series	4476 (26.38)
Combined precancerous lesions	
Low-grade dysplasia, oesophagus	682 (8.38)
High-grade dysplasia, oesophagus	251 (3.08)
Barrett oesophagus	34 (0.42)
Treatment method	
Surgery	998 (12.26)
Chemotherapy	1459 (17.93)
Radiotherapy	755 (9.28)
Concomitant chemo-radiation	1932 (23.74)
Stent insertion	921 (5.43)

To avoid confounding effects on mortality by pre-existing or combined other cancer, individuals with any diagnosis of cancer were excluded. Because the linkage with the database of the Korea Central Cancer Registry was unavailable due to strict privacy control law, the stage of EC could not be analysed. Instead, stent insertion was examined using the Korean National Health Insurance Services (NHIS) billing claims database for differentiating advanced EC.^10,11^

This study collected the data from the NCSP database, which included information on the participants’ demographic features. We collected the data from the National Health Insurance Service (NHIS). The need for informed consent for this specific study was waived by IRB review because the KNCSP database is quite large. With permission from the Ministry of Health and Welfare, the investigators used data maintained and deidentified by the NHIS. This study was approved by the Institutional Review Board of Gangnam Severance Hospital, Korea (Institutional Review Board no. 3-2017-0362).

### Outcomes

The primary outcome of this study was EC mortality. Information concerning the cause of death was obtained by linking the NCSP database and the death certificate database from Statistics Korea. EC mortality is defined as EC-specific death that the cause of death is EC. Furthermore, chronic diseases such as diabetes mellitus (DM), heart disease, cerebrovascular disease, liver disease, pulmonary disease or chronic kidney disease were adjusted using ICD-10 codes.

In this study, 16,969 EC patients diagnosed in 2007–2014 were followed until December 2015. During follow-up period, the association between the history of NSCP for gastric cancer and EC mortality was analysed.

### Exposure to screening test

In this study, data on exposure to the screening test were collected from the KNCSP database. The exposure to screening test was investigated from January 2002 to the date 6 months prior to being diagnosed with EC. Testing that within 6 months of being diagnosed with EC was not considered as exposure to the screening test because such tests had a diagnostic role rather than a screening role for EC. We defined ever-screened as having had at least one gastric cancer-screening test including upper endoscopy or UGIS during that period. The number of screening tests during the 5 years before diagnosis was evaluated. The time interval since the last screening was categorised as follows: 6–11 months, 12–23 months, 24–35 months and ≥36 months.

### Statistical analysis

The baseline data are expressed as means (SD) or numbers (%). The mortality rate (MR) per 1000 was measured considering the follow-up duration. To investigate the effects of gastric cancer-screening test on reducing mortality from EC, hazard ratios (HRs) with 95% confidence intervals (CIs) for dying from EC were obtained via Cox proportional-hazards regression analysis. We conducted subgroup analyses stratified by age, sex, income level, the last screening method, stent insertion and time interval since screening. HRs were adjusted by age, sex and underlying chronic disease using multiple Cox proportional hazard regression analysis. To compare mortality reduction between upper endoscopy and UGIS, Bonferroni post hoc analysis was used. Survival curves were estimated using the Kaplan–Meier analysis and log-rank test. *P*-values<0.05 were considered statistically significant, except Bonferroni post hoc analysis. The significant *P*-value for Bonferroni post hoc analysis was 0.05/3. All statistical analyses were conducted using SAS version 9.4 (SAS Institute, Cary, NC).

## Results

### Baseline characteristics of study cohort

Table 1 shows the baseline characteristics of the study cohort. Approximately half of the subjects (48.93%) had never been screened. Among ever-screened subjects, upper endoscopy or UGIS was similarly selected as the last screening method before diagnosis of EC. A total of 921 (5.43%) patients underwent stent insertion.

### Association between receipt of screening and mortality

Among all 16,969 EC patients, 6729 (39.65%) patients had EC-specific death during the follow-up period. The HRs of mortality according to the baseline characteristics including screening features are provided in Table 2. Older age, male, lower income level and stent insertion status were significantly associated with mortality.

**Table 2:** HRs and 95% CIs for the mortality rate according to baseline characteristics.

	*N*	Death	Duration	MR (per 1000)	Crude HR (95% CI)	*P*
Age (year)						<0.0001
<65	7610	2682	22,847.65	117.3863	1	
65–74	6399	2590	17,418.38	148.6935	1.233 (1.168–1.301)	
≥75	2960	1457	5560.98	262.004	1.956 (1.835–2.085)	
*Sex*						<0.0001
Male	15136	6230	40,391.13	154.2418	1	
Female	1833	499	5435.88	91.7975	0.634 (0.579–0.695)	
Income level						<0.0001
High	4203	1527	11,639.94	131.1862	1	
Middle	7526	2939	20,957.18	140.2383	1.075 (1.010–1.143)	
Low	5240	2263	13,229.88	171.0522	1.274 (1.194–1.359)	
Ever-screened						<0.0001
No	8303	3718	21,981.27	169.144	1	
Yes	8666	3011	23,845.73	126.27	0.708 (0.675–0.743)	
Number of screening during 5 years before diagnosis						
None	8303	3718	21,981.27	169.44	1	<0.0001
1	4542	1677	12,958.37	129.4144	0.748 (0.706–0.792)	
≥2 or over	4124	1334	10,887.36	122.5274	0.665 (0.624–0.708)	
Time interval since the last screening (month)						<0.0001
Never-screened	8303	3718	21,981.27	169.144	1	
6–11	1376	526	3836.04	137.1205	0.798 (0.728–0.874)	
12–23	3273	1144	9340.12	122.4823	0.694 (0.650–0.742)	
24–35	2460	765	6787.04	112.7148	0.623 (0.576–0.673)	
≥36 or over	1557	576	3882.53	148.357	0.806 (0.739–0.880)	
The last screening method						<0.0001
Never-screened	8303	3718	21,981.27	169.144	1	
Upper endoscopy	4190	1108	11,839.38	93.586	0.515 (0.481–0.550)	
Upper gastrointestinal series	4476	1903	12,006.35	158.4994	0.907 (0.858–0.959)	
Stent insertion						
No	16,048	6048	44,977.42	134.4675	1	<0.0001
Stent insertion	921	681	849.59	801.5666	5.211 (4.806–5.650)	

**Fig. 1:**
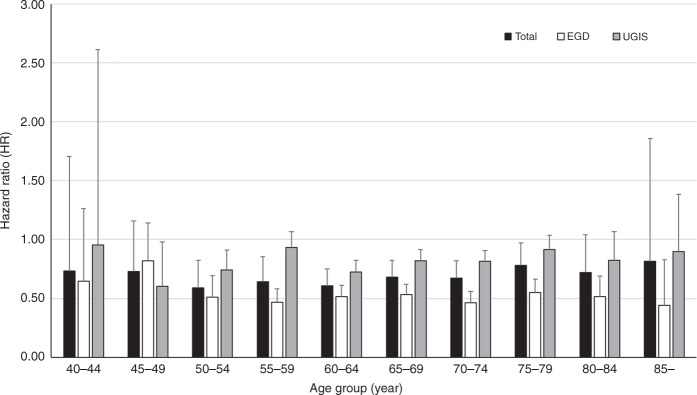
Association between screening and oesophageal cancer mortality according to the last screening modality since the last screening in comparison with never-screened individuals. Total indicates upper endoscopy or UGIS as the last screening modality. EGD upper endoscopy, UGIS upper gastrointestinal series.

When analysing the survival rate among never-screening, upper endoscopy and UGIS, upper endoscopy showed a survival benefit compared with never-screening, not UGIS (Supplement 1). Table 3 shows results of the multivariate analysis of the association between receipt of screening test and mortality. In Table 3, model 2 was adjusted by age, sex, income level and underlying chronic diseases. Compared with subjects who had never been screened, the overall HR for dying from EC among ever-screened subjects was 0.647 (95% CI, 0.617–0.679). According to the last screening modality, the HRs of death from EC were 0.497 (95% CI, 0.464–0.531) for upper endoscopy and 0.792 (95% CI, 0.749–0.838) for UGIS. The difference in HRs between upper endoscopy and UGIS were statistically significant (using Bonferroni method). Compared with never-screened individuals, those screened last by upper endoscopy consistently showed lower HRs for all age groups over 50 years; in contrast, UGIS did not (Fig. 1). As the number of screening tests performed per subject increased, the HRs of death from EC decreased: 0.703 (95% CI, 0.663–0.744) and 0.588 (95% CI, 0.552–0.626) for one and ≥2 times, respectively (Table 3).

**Table 3:** Association between receipt of screening test and mortality.

	*N*	Death	Duration	MR (per 1000)	Model 1	*P*	Model 2	*P*
Ever-screened						<0.0001		<0.0001
No	8303	3718	21,981.27	169.144	1		1	
Yes	8666	3011	23,845.73	126.27	0.708 (0.675–0.743)		0.647 (0.617–0.679)	
Number of screening during 5 years before diagnosis						<0.0001		<0.0001
None	8303	3718	21,981.27	169.44	1		1	
1	4542	1677	12,958.37	129.4144	0.748 (0.706–0.792)		0.703 (0.663–0.744)	
≥2 or over	4124	1334	10,887.36	122.5274	0.665 (0.624–0.708)		0.588 (0.552–0.626)	
Time interval since the last screening (month)						<0.0001		<0.0001
No	8303	3718	21,981.27	169.144	1		1	
6–11	1376	526	3836.04	137.1205	0.798 (0.728–0.874)		0.731 (0.667–0.801)	
12–23	3273	1144	9340.12	122.4823	0.694 (0.650–0.742)		0.635 (0.594–0.679)	
24–35	2460	765	6787.04	112.7148	0.623 (0.576–0.673)		0.564 (0.522–0.610)	
≥36 or over	1557	576	3882.53	148.357	0.806 (0.739–0.880)		0.742 (0.679–0.810)	
The last screening method^a^						<0.0001		<0.0001
Never-screened	8303	3718	21,981.27	169.144	1		1	
Upper endoscopy	4190	1108	11,839.38	93.586	0.515 (0.481–0.550)		0.497 (0.464–0.531)	
Upper gastrointestinal series	4476	1903	12,006.35	158.4994	0.907 (0.858–0.959)		0.792 (0.749–0.838)	

Model 1: crude.

Model 2: adjusted by age, sex, income level and underlying chronic disease, including diabetes mellitus, heart disease, cerebrovascular disease, liver disease, pulmonary disease or chronic kidney disease.

^a^When multiple comparison test using Bonferroni method was performed, all *P*-values of ‘never-screened vs. upper endoscopy’, ‘never-screened vs. upper gastrointestinal series’ and ‘upper endoscopy vs. upper gastrointestinal series’ were <0.0001 in both model 1 and model 2.

According to the time interval since screening, the HRs of death from EC were 0.731 (95% CI, 0.667–0.801) for 6–11 months, 0.635 (95% CI, 0.594–0.679) for 12–23 months, 0.564 (95% CI, 0.522–0.610) for 24–35 months, and 0.742 (95% CI, 0.679–0.810) for ≥36 months (Table 3). This indicates that the current biennial screening in KNCSP is effective to reduce the mortality of EC subjects.

Table 4 shows the association between receipt of screening test and mortality, for individuals <75 years and ≥75 years. Compared with never-screened subjects, individuals ≥75 years had, which is different from a previous report about gastric cancer.^6^

**Table 4:** Association between receipt of screening test and mortality between <75 years and ≥75 years.

	<75 year								≥75 years			
	*N*	Death	Duration	MR (per 1000)	Model 2	*P*	*N*	Death	Duration	MR (per 1000)	Model 2	*P*
Ever-screened						<0.0001						<0.0001
No	6889	2961	19,453.64	152.208	1		1414	757	2527.63	299.49	1	
Yes	7120	2311	20812.38	111.0397	0.643 (0.608–0.680)		1546	700	3033.35	230.7677	0.761 (0.685–0.846)	
Number of screening during 5 years before diagnosis						<0.0001						<0.0001
None	6889	2961	19,453.64	152.208	1		1414	757	2527.63	299.49	1	
1	3770	1309	11,429.17	114.5315	0.696 (0.652–0.743)		772	368	1529.2	240.6487	0.813 (0.716–0.922)	
≥2 or over	3350	1002	9383.21	106.7865	0.583 (0.542–0.627)		774	332	1504.15	220.7222	0.710 (0.622–0.811)	
Time interval since the last screening (month)						<0.0001						<0.0001
No	6889	2961	19,453.64	152.208	1		1414	757	2527.63	299.49	1	
6–11	1121	400	3360.68	119.0236	0.714 (0.643–0.793)		255	126	475.36	265.0598	0.900 (0.743–1.090)	
12–23	2747	915	8230.73	111.1688	0.649 (0.602–0.699)		526	229	1109.4	206.4184	0.681 (0.586–0.791)	
24–35	2017	575	5902.29	97.4199	0.551 (0.503–0.603)		443	190	884.75	214.7491	0.715 (0.609–0.841)	
≥36 or over	1235	421	3318.69	126.8574	0.723 (0.653–0.801)		322	155	563.84	274.9015	0.873 (0.733–1.039)	
The last screening method^a^						<0.0001						<0.0001
Never-screened	6889	2961	19,453.64	152.208	1		1414	757	2527.63	299.49	1	
Upper endoscopy	3687	936	10,766.38	86.93727	0.505 (0.469–0.544)		503	172	1072.99	160.299	0.539 (0.456–0.638)	
Upper gastrointestinal series	3433	1375	10,045.99	136.8705	0.799 (0.748–0.853)		1043	528	1960.36	269.3384	0.878 (0.784–0.984)	

Model 1: crude.

Model 2: adjusted by age, sex, income level and underlying chronic disease, including diabetes mellitus, heart disease, cerebrovascular disease, liver disease, pulmonary disease or chronic kidney disease.

^a^When multiple comparison test using Bonferroni method was performed, all *P*-values of ‘never-screened vs. upper endoscopy’, ‘never-screened vs. upper gastrointestinal series’ and ‘upper endoscopy vs. upper gastrointestinal series’ were <0.0001 in both model 1 and model 2.

## Discussion

According to our study, the current NCSP for gastric cancer was effective in reducing the mortality of EC, and upper endoscopy was superior to UGIS.

In our cohort of EC, the portion of ever-screened subjects was ~50%, which is similar to that of general population.^12,13^ In this study, the rate of upper endoscopy and UGIS performed as the last screening was similar. This result contradicts results of previous studies indicating that the portion of patients screened by upper endoscopy is increasing.^5,14^ A likely reason for this contradictory result is that we could not consider the opportunistic screening by individuals; thus, subjects who underwent UGIS as part of their NCSP screening might be undergone upper endoscopy as opportunistic screening. This may overestimate the effect of UGIS in NCSP on the reduction of EC mortality.

Our study showed that the NCSP for gastric cancer can reduce EC mortality by 35.3%. Furthermore, according to the last screening modality, the HR of death from EC for upper endoscopy was significantly lower than that for UGIS (0.497 vs. 0.792). Univariate analysis showed that upper endoscopy screening, but not UGIS, was beneficial to survival compared with no-screening. However, multivariate analysis showed that UGIS had an effect on reducing EC mortality, but this effect was significantly weaker than that of upper endoscopy. Furthermore, upper endoscopy showed a consistent reduction of EC mortality in all age groups over 50 years, whereas UGIS did not. Thus, to reduce EC mortality, upper endoscopy can be more effective than UGIS in NCSP for gastric cancer.

A recent study concerning the effectiveness of NCSP showed that gastric cancer mortality was reduced only in individuals 40–74 years, not 75 years or over.^6^ However, in our study, upper endoscopy consistently reduced EC mortality in all age groups since 50 years. A comparison of individuals <75 and ≥75-years old showed that the national cancer screening within 36 months could also reduce EC mortality in individuals ≥75 years. In fact, 17.4% of the EC patients were ≥75 years old in our cohort. This indicates that the national cancer screening can be also effective in reducing EC mortality in the elderly.

According to our data, all time intervals since the last screening (6–11, 12–23, 24–35, 36 months over) could reduce EC mortality compared with never-screened. That is, this suggested that screening itself may be important to decrease mortality in EC, similar to the previous China  study.^15^ The previous study showed one-time endoscopic screening and then intervention including endoscopic resection or coagulation for dysplasia/early cancer significantly reduced mortality caused by EC. Precancerous lesions such as dysplasia was observed in about 11.9%. However, endoscopic submucosal dissection was covered by insurance since November 2018 in Korea, so we could not analyse whether the lesions were resected or not. Thus, the preventive effect of EC by intervention of precursor lesions was not evaluated. In our study, risk reduction of EC mortality was not correlated with degree of time intervals since the last screening, that is, the risk of EC mortality was not decreased according to shorter time intervals since the last screening. The reason was probably due to misclassification of upper endoscopy and UGIS as screening tests. That is, those with symptoms or signs suspicious for EC could have undergone screening test. To prevent misclassification in our study, testing that within 6 months of being diagnosed with EC was not considered as exposure to the screening test because such tests had a diagnostic role rather than a screening role for EC. In spite of that, 6–11 months since the last screening showed higher HR for dying from EC than 12–35 months since the last screening.

Oesophageal squamous cell carcinoma and adenocarcinoma are the two most common histopathological types of EC. Oesophageal squamous cell carcinoma remains the dominant type of EC worldwide, particular in East Asia, including Korea.^16,17^ The percentage of oesophageal adenocarcinoma amongst oesophageal malignancies was as low as 1–4% in Korea in a previous study.^18^ Endoscopy is known to be useful in screening for Barrett-related adenocarcinomas worldwide, but the usefulness of screening for squamous cell carcinoma is not yet known.^18,19^ For Barrett’s oesophagus and early adenocarcinoma, active endoscopic screening with adequate surveillance is recommended for people with chronic reflux symptoms and at least one risk factor for oesophageal adenocarcinoma, depending on the lesion found on the index endoscopy and pathology.^18^

However, there is no standardised screening programme to detect precancerous lesions such as squamous dysplasia of squamous cell carcinoma.^18,20^ In Japan, controversy exists concerning whether precancerous lesions should be actively detected by screening.^18^ In China, however, which has a high incidence of EC, endoscopic screening of EC is considered to be cost-effective in high-risk areas.^21^ In China, the following two key measures to combat oesophageal squamous cell carcinoma were recommended as cost-effective programmes, considering the acceptance of the population and the distribution of wealth in different regions:^18,21^ (1) screening once beginning at the age of 50, following up 5 years after detecting low-grade dysplasia and 3 years after intermediate-grade dysplasia, for areas with limited access to healthcare, impoverished areas and areas in which it is difficult to track the target population economy;^18,21^ and (2) screening three times beginning at the age of 40, and monitoring low-grade dysplasia and intermediate-grade dysplasia as above, for areas with appropriate access to healthcare, and economies that are more advanced and areas in which there are good monitoring programmes of the target population.^18,21^

In Korea, the incidence of EC is increasing, as shown in our study and similar to a previous study.^9^ However, the incidence of EC is not high, so it is difficult to determine the necessity or strategy of a screening programme for EC like in China. However, a screening strategy should be investigated in the future for high-risk groups such as old age, smoking or patients with head and neck cancer.

Instead of screening programme for EC, there is nationwide screening programme for gastric cancer in Korea. Because examinations for gastric cancer can also detect EC, screening programmes for gastric cancer could be effective in reducing EC mortality. If possible, the nationwide screening programme for gastric cancer should be re-evaluated for its effectiveness to reduce the mortality of both gastric cancer and EC. According to our data, the current NCSP of biennial upper endoscopic screening for gastric cancer is also effective in reducing the mortality of EC, especially for ≥50-year-old individuals. This suggests that NCSP, which targets gastric cancer, can be expected to have an additional survival benefit for not only gastric cancer but also EC. However, NCSP was designed for gastric cancer, but not for  EC. Thus, screening programme for EC should be investigated in the future. In addition, a cost-analysis for person-life saved by enrolment in this screening programme is important to confirm the screening effect for EC. Thus, a cost-analysis should be performed in the future.

This study had several limitations. First was the self-selection bias. The participants in the NCSP might be overrepresented by healthy or health-conscious individuals.^6^ This could have led to an overestimation of the effectiveness of the screening programme. In our data, the HR of death from EC of UGIS was lower than that of no-screening. Considering the method itself, UGIS is a less effective method to examine the oesophagus. This result may be influenced by the fact that participants in the NCSP might be more health conscious. The self-selection bias can be related to detection bias in our study. The subjects with chronic diseases may be different from others in terms of health-consciousness or hospital visits. When adjusted by age, sex, income and underlying chronic diseases, the effect of screening on reducing EC mortality was also observed in Tables 3 and 4. Therefore, the self-selection and detection bias may not affect significantly our results. The length bias is one of the issues for screening studies. However, the length bias can be more related to slowly growing tumours, so it may not be important for EC.

Second was the lead time bias. Ever-screened patients may seem to be surviving longer solely because they are diagnosed earlier, not screening-test effect. However, according to our data, the survival curves showed a plateau at almost 70% for EGD, whereas almost 40% for UGIS. These survival curves mean that the favourable results are not solely due to lead time bias.

Third, opportunistic screening in Korea was not considered in this study. Considering opportunistic screening, the effect of upper endoscopy on reducing EC mortality would have been greater.

Fourth limitation is that a lack of data regarding the specific details such as specific symptoms, such as oesophageal reflux, *Helicobacter pylori* status, histologic differentiation of EC or cancer stage, because we collected the data from the NHIS with permission from the Ministry of Health and Welfare. In addition, it is impossible to link the NHIS database and the National Cancer Registry because of the Personal Information Protection Act in Korea.

In conclusion, the current NCSP for gastric cancer was effective in reducing the mortality of EC, and upper endoscopy was superior to UGIS. Thus, we can improve the prognosis of EC through the nationwide upper endoscopic screening policy for gastric cancer. This may suggest that endoscopic screening for EC can be reasonable in high-risk subjects.

## Supplementary information

Supplement Fig. 1

Supplement Fig. 1 legend

## Data Availability

The data sets used and/or analysed during this study are available from the corresponding author on reasonable request.
