# Correction: Association between the National Cancer Screening Programme (NSCP) for gastric cancer and oesophageal cancer mortality

**DOI:** 10.1038/s41416-020-0946-z

**Published:** 2020-06-17

**Authors:** Jie-Hyun Kim, Kyung-Do Han, Jung Kuk Lee, Hyun-Soo Kim, Jae Myung Cha, Sohee Park, Joo Sung Kim, Won Ho Kim, for the Big Data Research Group (BDRG) of the Korean Society of Gastroenterology (KSG)

**Affiliations:** 1 Department of Internal Medicine, Yonsei University College of Medicine, Seoul, Korea; 2 Department of Biostatistics, The Catholic University, Seoul, Korea; 3 Department of Biostatistics, Yonsei University Wonju College of Medicine, Wonju, Korea; 4 Department of Internal Medicine, Yonsei University Wonju College of Medicine, Wonju, Korea; 5 Department of Internal Medicine, Kyung Hee University School of Medicine, Seoul, Korea; 6 Department of Biostatistics, Graduate School of Public Health, Yonsei University, Seoul, Korea; 7 Department of Internal Medicine, Seoul National University School of Medicine, Seoul, Korea

**Keywords:** Population screening, Cancer screening

Correction to: *British Journal of Cancer* (2020); 10.1038/s41416-020-0883-x, published online 13 May 2020

Since the publication of this paper, the authors have noticed an error in the name and department of author Sohee Park. Dr. Park’s name was incorrectly listed as So Hee Park. The Department of Biostatistics, Graduate School of Public Health was incorrectly listed as the Department of Public Health, Graduate School. Both errors have been corrected above. The authors apologise for this oversight.

