# Supplementary figures and images for: Association between the National Cancer Screening Programme (NSCP) for gastric cancer and oesophageal cancer mortality
Source: Br J Cancer. 2020 May 13;123(3):480–6. doi: 10.1038/s41416-020-0883-x (PMC7403142; doi:10.1038/s41416-020-0883-x)

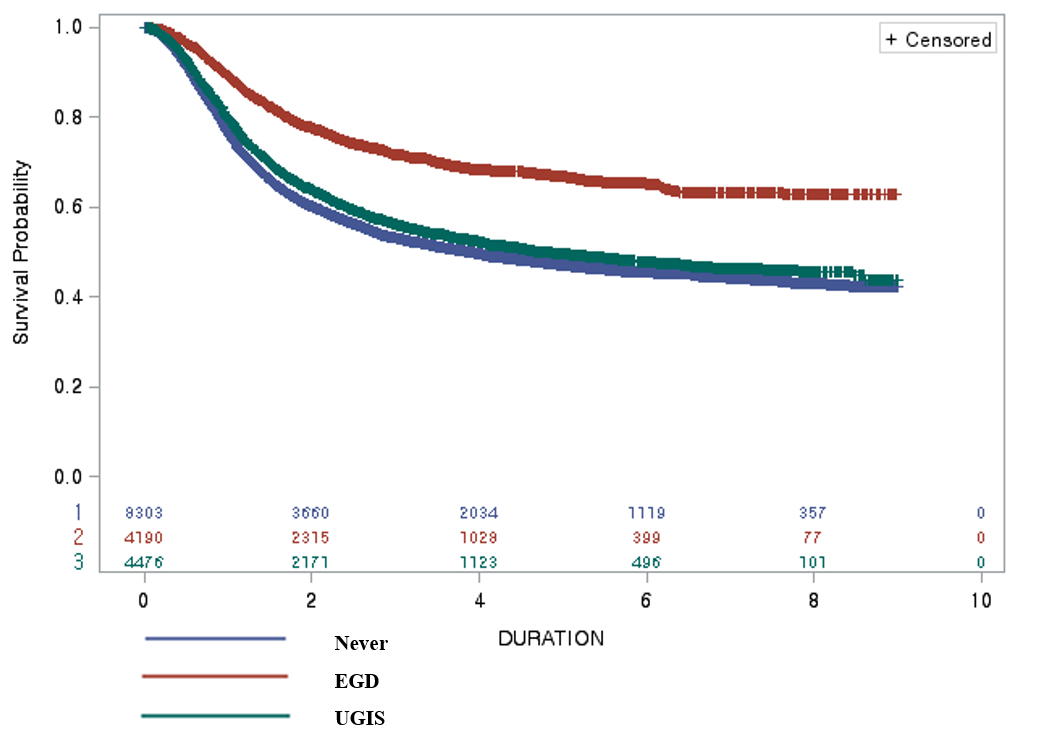

Supplement: Supplementary file 1 — Supplement Fig. 1 [file 41416_2020_883_MOESM1_ESM.tif]
