# Supplementary material for: Association between the National Cancer Screening Programme (NSCP) for gastric cancer and oesophageal cancer mortality
Source: Br J Cancer. 2020 May 13;123(3):480–6. doi: 10.1038/s41416-020-0883-x (PMC7403142; doi:10.1038/s41416-020-0883-x)
Supplement: Supplementary file 2 — Supplement Fig. 1 legend [file 41416_2020_883_MOESM2_ESM.docx]

**Supplement Fig 1.** Survival effect according to no-screening (never), upper gastrointestinal series (UGIS), and upper endoscopy (EGD) in oesophageal cancer.
